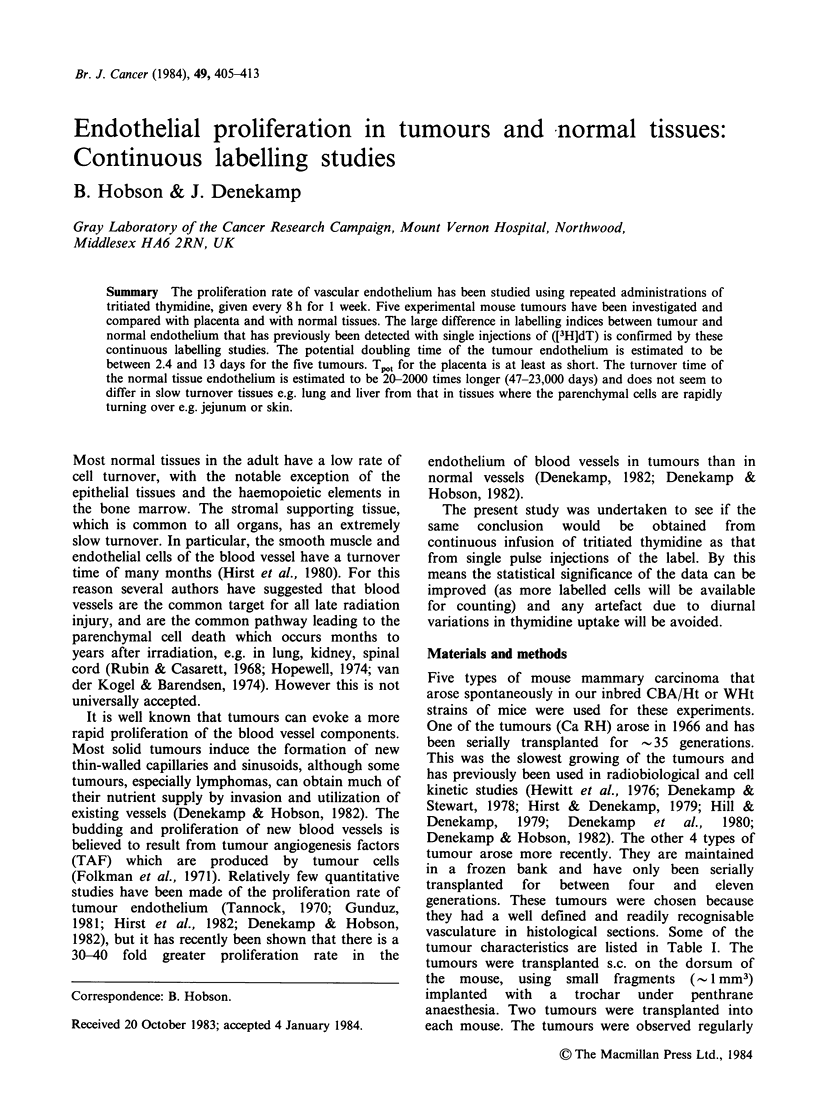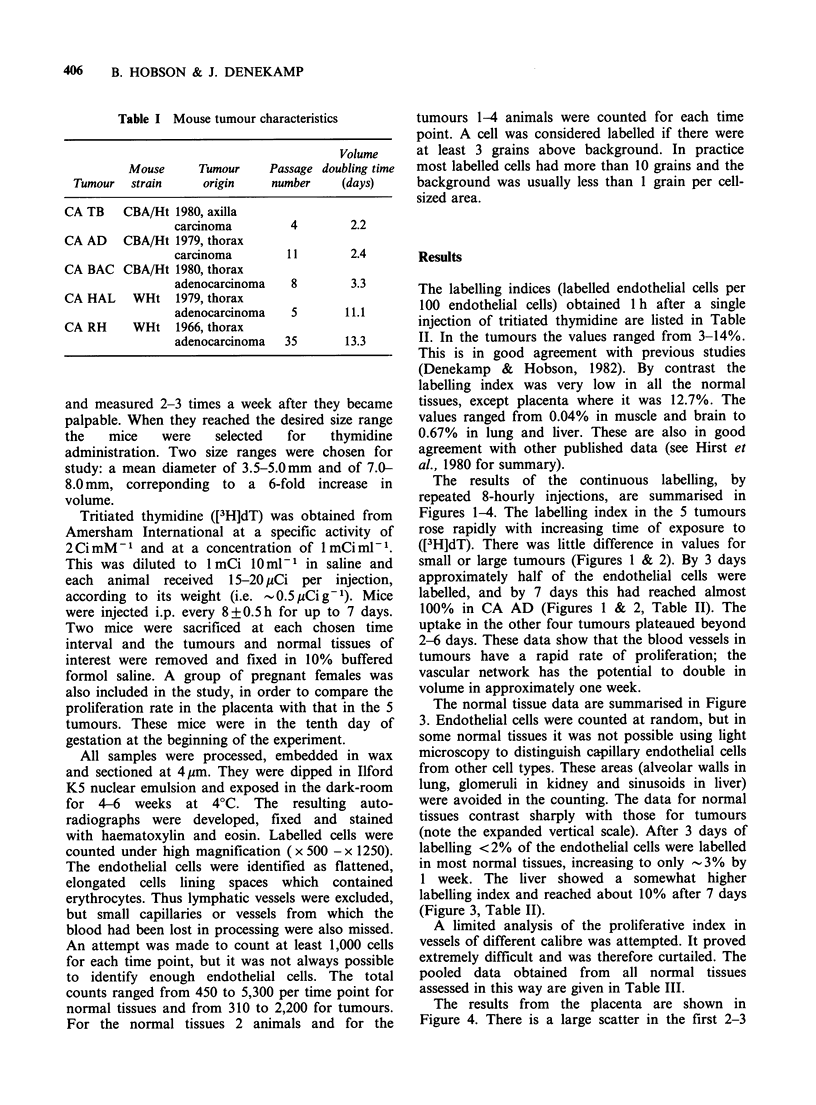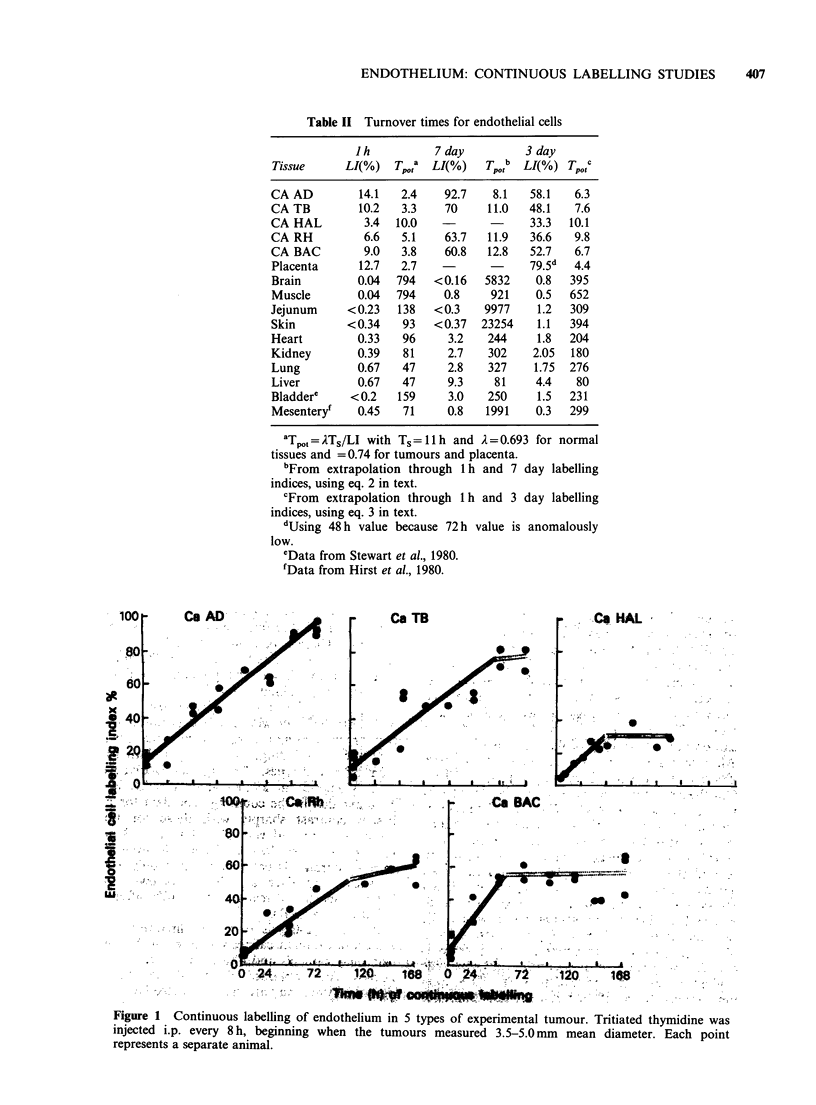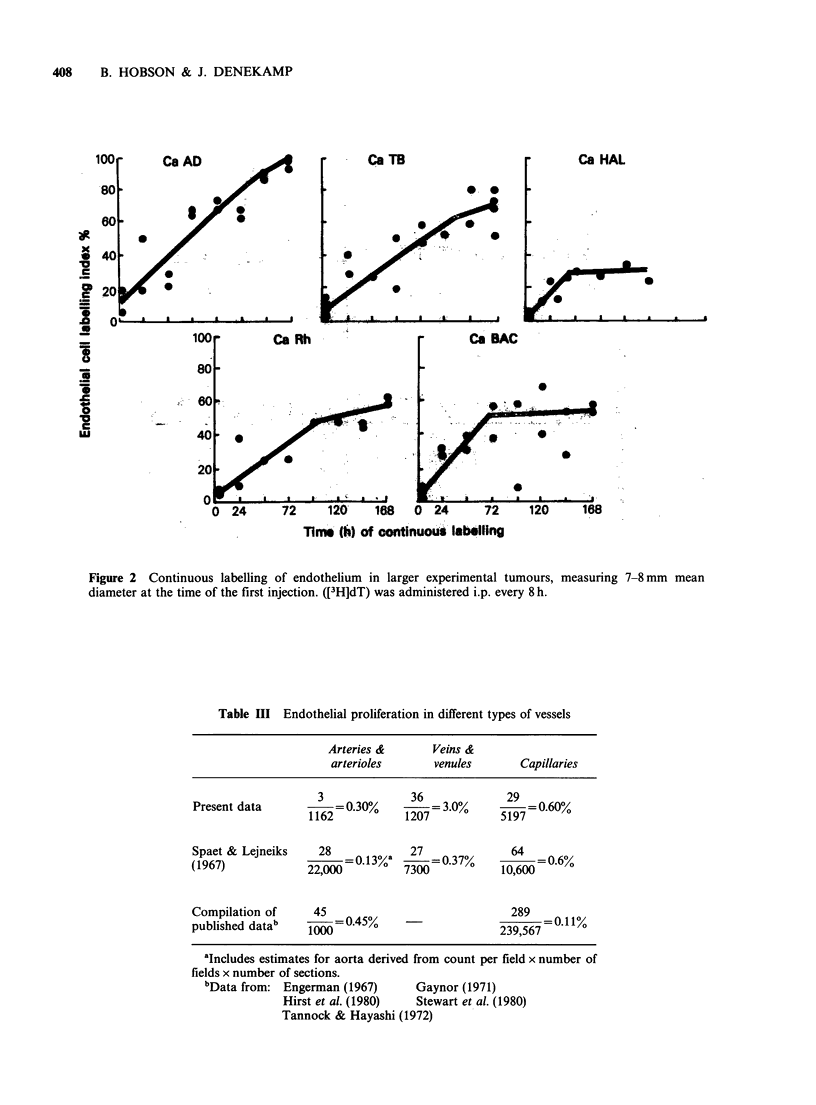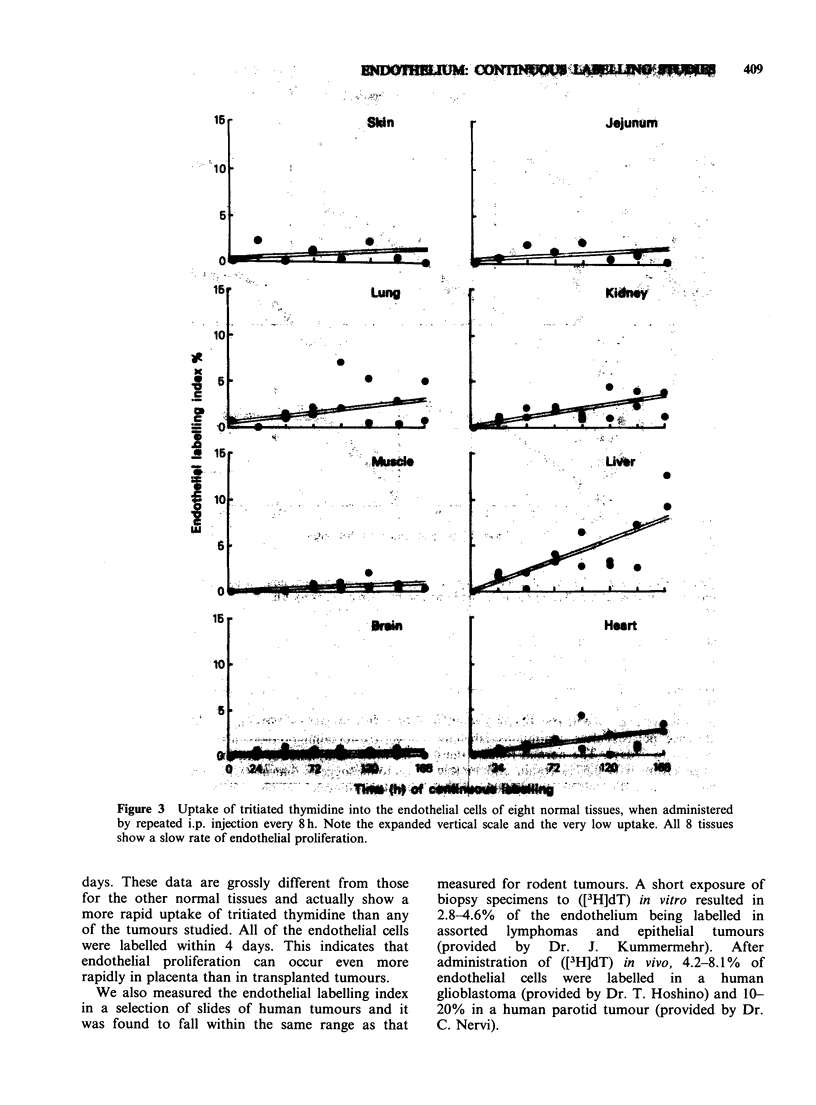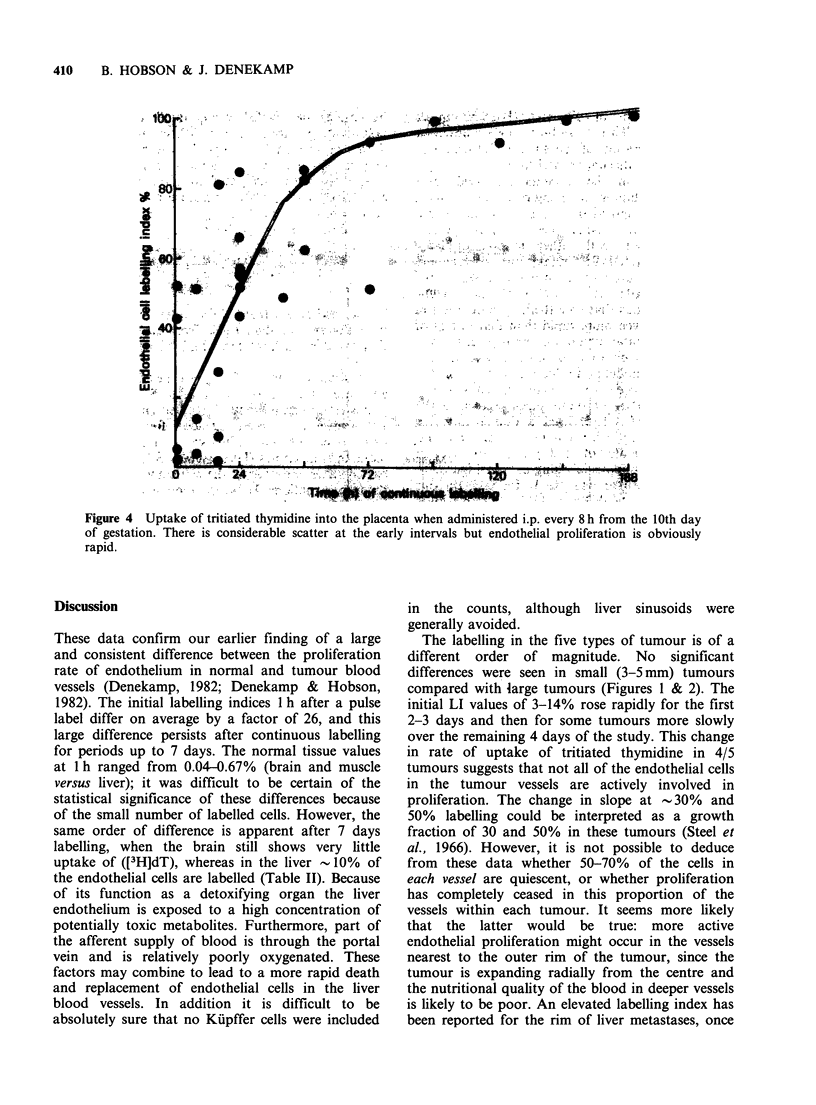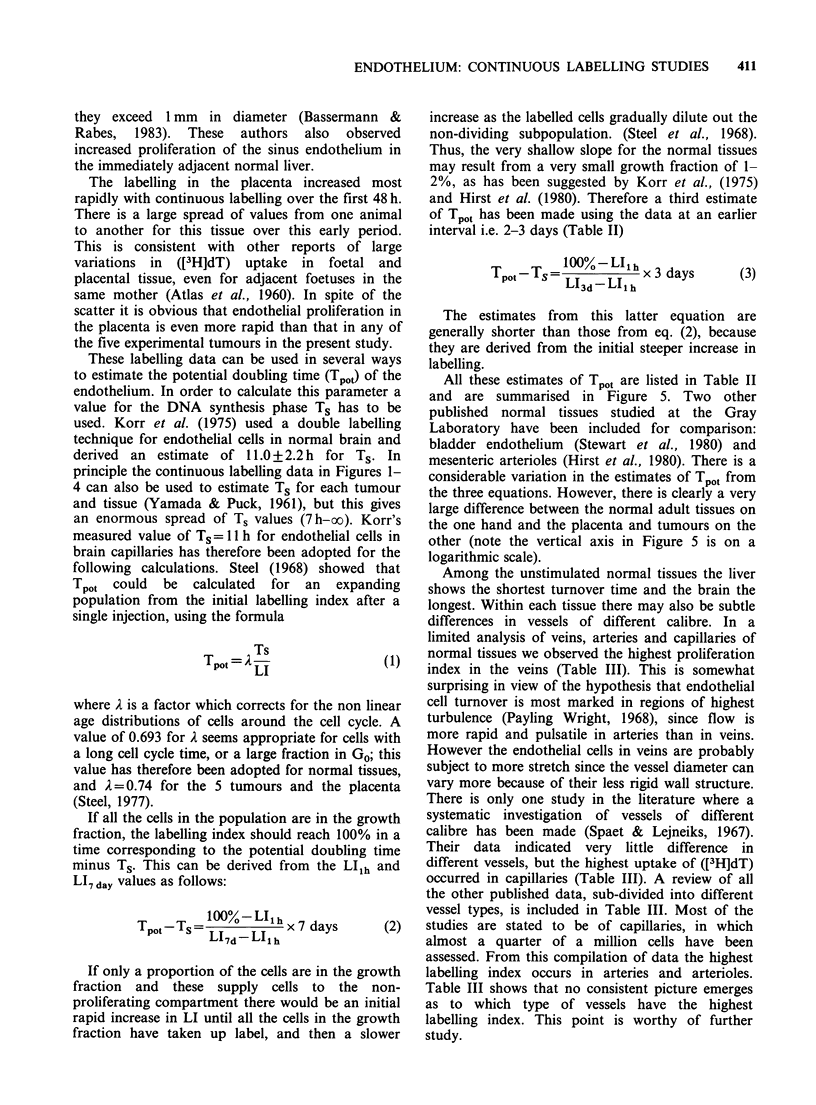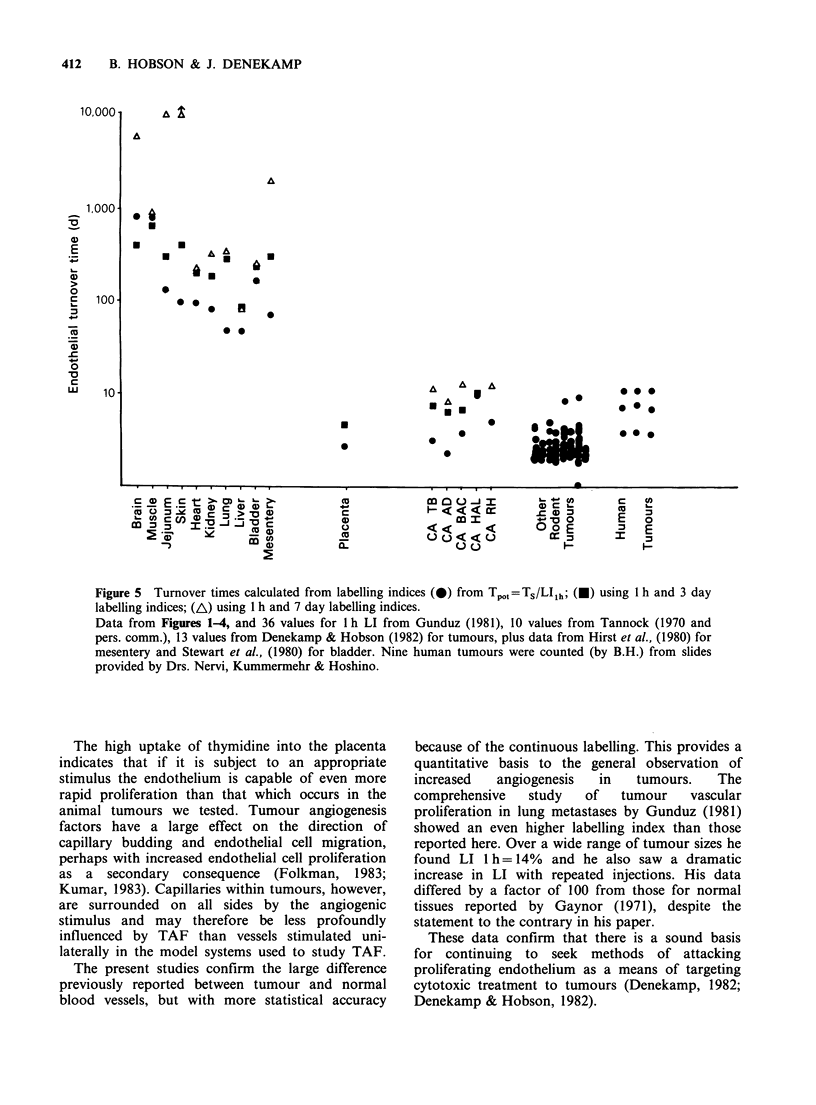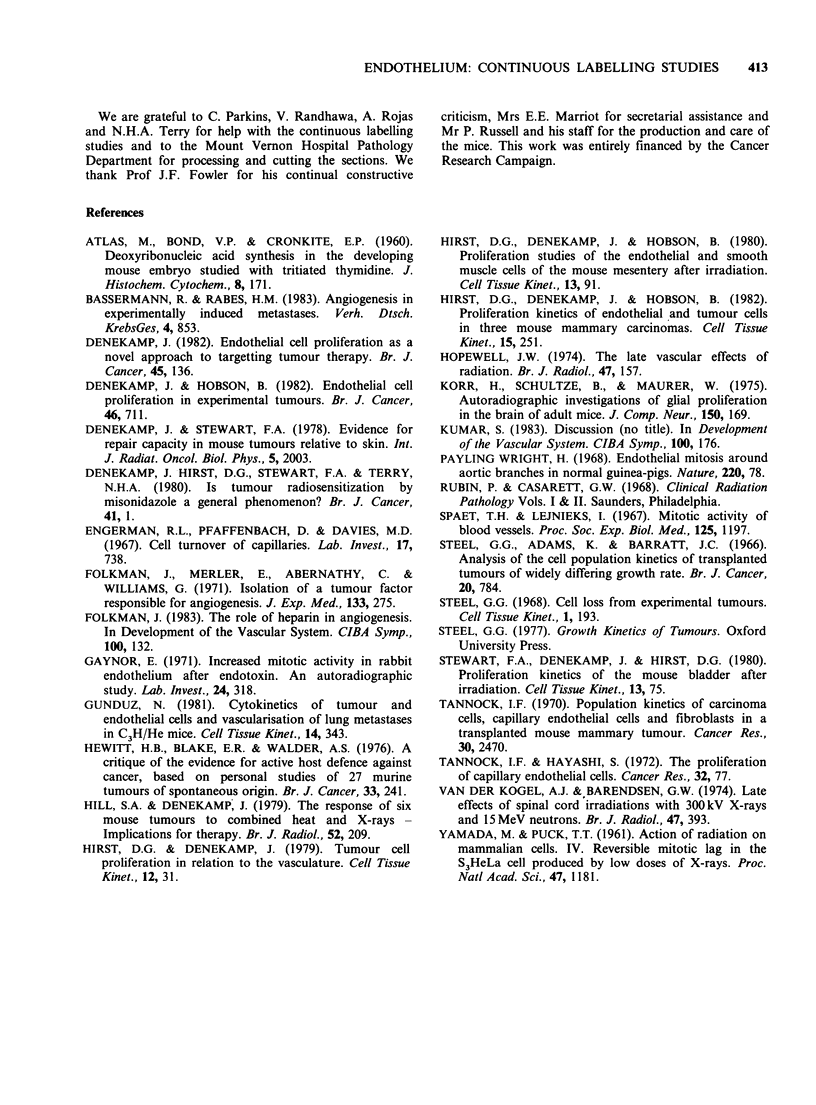# Endothelial proliferation in tumours and normal tissues: continuous labelling studies.

**DOI:** 10.1038/bjc.1984.66

**Published:** 1984-04

**Authors:** B. Hobson, J. Denekamp

## Abstract

The proliferation rate of vascular endothelium has been studied using repeated administrations of tritiated thymidine, given every 8 h for 1 week. Five experimental mouse tumours have been investigated and compared with placenta and with normal tissues. The large difference in labelling indices between tumour and normal endothelium that has previously been detected with single injections of ([3H]dT) is confirmed by these continuous labelling studies. The potential doubling time of the tumour endothelium is estimated to be between 2.4 and 13 days for the five tumours. Tpot for the placenta is at least as short. The turnover time of the normal tissue endothelium is estimated to be 20-2000 times longer (47-23,000 days) and does not seem to differ in slow turnover tissues e.g. lung and liver from that in tissues where the parenchymal cells are rapidly turning over e.g. jejunum or skin.


*Br. J. Cancer* (1984), **49**, 405–413

### Endothelial proliferation in tumours and normal tissues: Continuous labelling studies

B. Hobson & J. Denekamp

*Gray Laboratory of the Cancer Research Campaign, Mount Vernon Hospital, Northwood, Middlesex HA6 2RN, UK*

**Summary** The proliferation rate of vascular endothelium has been studied using repeated administrations of tritiated thymidine, given every 8 h for 1 week. Five experimental mouse tumours have been investigated and compared with placenta and with normal tissues. The large difference in labelling indices between tumour and normal endothelium that has previously been detected with single injections of ([$^3$H]dT) is confirmed by these continuous labelling studies. The potential doubling time of the tumour endothelium is estimated to be between 2.4 and 13 days for the five tumours. $T_{pot}$ for the placenta is at least as short. The turnover time of the normal tissue endothelium is estimated to be 20–2000 times longer (47–23,000 days) and does not seem to differ in slow turnover tissues e.g. lung and liver from that in tissues where the parenchymal cells are rapidly turning over e.g. jejunum or skin.

Most normal tissues in the adult have a low rate of cell turnover, with the notable exception of the epithelial tissues and the haemopoietic elements in the bone marrow. The stromal supporting tissue, which is common to all organs, has an extremely slow turnover. In particular, the smooth muscle and endothelial cells of the blood vessel have a turnover time of many months (Hirst *et al.*, 1980). For this reason several authors have suggested that blood vessels are the common target for all late radiation injury, and are the common pathway leading to the parenchymal cell death which occurs months to years after irradiation, e.g. in lung, kidney, spinal cord (Rubin & Casarett, 1968; Hopewell, 1974; van der Kogel & Barendsen, 1974). However this is not universally accepted.

It is well known that tumours can evoke a more rapid proliferation of the blood vessel components. Most solid tumours induce the formation of new thin-walled capillaries and sinusoids, although some tumours, especially lymphomas, can obtain much of their nutrient supply by invasion and utilization of existing vessels (Denekamp & Hobson, 1982). The budding and proliferation of new blood vessels is believed to result from tumour angiogenesis factors (TAF) which are produced by tumour cells (Folkman *et al.*, 1971). Relatively few quantitative studies have been made of the proliferation rate of tumour endothelium (Tannock, 1970; Gunduz, 1981; Hirst *et al.*, 1982; Denekamp & Hobson, 1982), but it has recently been shown that there is a 30–40 fold greater proliferation rate in the endothelium of blood vessels in tumours than in normal vessels (Denekamp, 1982; Denekamp & Hobson, 1982).

The present study was undertaken to see if the same conclusion would be obtained from continuous infusion of tritiated thymidine as that from single pulse injections of the label. By this means the statistical significance of the data can be improved (as more labelled cells will be available for counting) and any artefact due to diurnal variations in thymidine uptake will be avoided.

### Materials and methods

Five types of mouse mammary carcinoma that arose spontaneously in our inbred CBA/Ht or WHt strains of mice were used for these experiments. One of the tumours (Ca RH) arose in 1966 and has been serially transplanted for ~35 generations. This was the slowest growing of the tumours and has previously been used in radiobiological and cell kinetic studies (Hewitt *et al.*, 1976; Denekamp & Stewart, 1978; Hirst & Denekamp, 1979; Hill & Denekamp, 1979; Denekamp *et al.*, 1980; Denekamp & Hobson, 1982). The other 4 types of tumour arose more recently. They are maintained in a frozen bank and have only been serially transplanted for between four and eleven generations. These tumours were chosen because they had a well defined and readily recognisable vasculature in histological sections. Some of the tumour characteristics are listed in Table I. The tumours were transplanted s.c. on the dorsum of the mouse, using small fragments (~1 mm$^3$) implanted with a trochar under penthrane anaesthesia. Two tumours were transplanted into each mouse. The tumours were observed regularly

Correspondence: B. Hobson.

Received 20 October 1983; accepted 4 January 1984.

© The Macmillan Press Ltd., 1984

**Table I** Mouse tumour characteristics

| *Tumour* | *Mouse strain* | *Tumour origin* | *Passage number* | *Volume doubling time (days)* |
|---|---|---|---|---|
| CA TB | CBA/Ht | 1980, axilla carcinoma | 4 | 2.2 |
| CA AD | CBA/Ht | 1979, thorax carcinoma | 11 | 2.4 |
| CA BAC | CBA/Ht | 1980, thorax adenocarcinoma | 8 | 3.3 |
| CA HAL | WHt | 1979, thorax adenocarcinoma | 5 | 11.1 |
| CA RH | WHt | 1966, thorax adenocarcinoma | 35 | 13.3 |

and measured 2–3 times a week after they became palpable. When they reached the desired size range the mice were selected for thymidine administration. Two size ranges were chosen for study: a mean diameter of 3.5–5.0 mm and of 7.0–8.0 mm, correponding to a 6-fold increase in volume.

Tritiated thymidine ([$^3$H]dT) was obtained from Amersham International at a specific activity of 2 Ci mM$^{-1}$ and at a concentration of 1 mCi ml$^{-1}$. This was diluted to 1 mCi 10 ml$^{-1}$ in saline and each animal received 15–20 $\mu$Ci per injection, according to its weight (i.e. $\sim$0.5 $\mu$Ci g$^{-1}$). Mice were injected i.p. every $8 \pm 0.5$ h for up to 7 days. Two mice were sacrificed at each chosen time interval and the tumours and normal tissues of interest were removed and fixed in 10% buffered formol saline. A group of pregnant females was also included in the study, in order to compare the proliferation rate in the placenta with that in the 5 tumours. These mice were in the tenth day of gestation at the beginning of the experiment.

All samples were processed, embedded in wax and sectioned at 4 $\mu$m. They were dipped in Ilford K5 nuclear emulsion and exposed in the dark-room for 4–6 weeks at 4°C. The resulting autoradiographs were developed, fixed and stained with haematoxylin and eosin. Labelled cells were counted under high magnification ($\times$500 – $\times$1250). The endothelial cells were identified as flattened, elongated cells lining spaces which contained erythrocytes. Thus lymphatic vessels were excluded, but small capillaries or vessels from which the blood had been lost in processing were also missed. An attempt was made to count at least 1,000 cells for each time point, but it was not always possible to identify enough endothelial cells. The total counts ranged from 450 to 5,300 per time point for normal tissues and from 310 to 2,200 for tumours. For the normal tissues 2 animals and for the tumours 1–4 animals were counted for each time point. A cell was considered labelled if there were at least 3 grains above background. In practice most labelled cells had more than 10 grains and the background was usually less than 1 grain per cell-sized area.

### Results

The labelling indices (labelled endothelial cells per 100 endothelial cells) obtained 1 h after a single injection of tritiated thymidine are listed in Table II. In the tumours the values ranged from 3–14%. This is in good agreement with previous studies (Denekamp & Hobson, 1982). By contrast the labelling index was very low in all the normal tissues, except placenta where it was 12.7%. The values ranged from 0.04% in muscle and brain to 0.67% in lung and liver. These are also in good agreement with other published data (see Hirst *et al.*, 1980 for summary).

The results of the continuous labelling, by repeated 8-hourly injections, are summarised in Figures 1–4. The labelling index in the 5 tumours rose rapidly with increasing time of exposure to ([$^3$H]dT). There was little difference in values for small or large tumours (Figures 1 & 2). By 3 days approximately half of the endothelial cells were labelled, and by 7 days this had reached almost 100% in CA AD (Figures 1 & 2, Table II). The uptake in the other four tumours plateaued beyond 2–6 days. These data show that the blood vessels in tumours have a rapid rate of proliferation; the vascular network has the potential to double in volume in approximately one week.

The normal tissue data are summarised in Figure 3. Endothelial cells were counted at random, but in some normal tissues it was not possible using light microscopy to distinguish capillary endothelial cells from other cell types. These areas (alveolar walls in lung, glomeruli in kidney and sinusoids in liver) were avoided in the counting. The data for normal tissues contrast sharply with those for tumours (note the expanded vertical scale). After 3 days of labelling <2% of the endothelial cells were labelled in most normal tissues, increasing to only $\sim$3% by 1 week. The liver showed a somewhat higher labelling index and reached about 10% after 7 days (Figure 3, Table II).

A limited analysis of the proliferative index in vessels of different calibre was attempted. It proved extremely difficult and was therefore curtailed. The pooled data obtained from all normal tissues assessed in this way are given in Table III.

The results from the placenta are shown in Figure 4. There is a large scatter in the first 2–3

**Table II** Turnover times for endothelial cells

| *Tissue* | *1 h LI(%)* | $T_{pot}{}^{a}$ | *7 day LI(%)* | $T_{pot}{}^{b}$ | *3 day LI(%)* | $T_{pot}{}^{c}$ |
|---|---|---|---|---|---|---|
| CA AD | 14.1 | 2.4 | 92.7 | 8.1 | 58.1 | 6.3 |
| CA TB | 10.2 | 3.3 | 70 | 11.0 | 48.1 | 7.6 |
| CA HAL | 3.4 | 10.0 | — | — | 33.3 | 10.1 |
| CA RH | 6.6 | 5.1 | 63.7 | 11.9 | 36.6 | 9.8 |
| CA BAC | 9.0 | 3.8 | 60.8 | 12.8 | 52.7 | 6.7 |
| Placenta | 12.7 | 2.7 | — | — | 79.5[d] | 4.4 |
| Brain | 0.04 | 794 | <0.16 | 5832 | 0.8 | 395 |
| Muscle | 0.04 | 794 | 0.8 | 921 | 0.5 | 652 |
| Jejunum | <0.23 | 138 | <0.3 | 9977 | 1.2 | 309 |
| Skin | <0.34 | 93 | <0.37 | 23254 | 1.1 | 394 |
| Heart | 0.33 | 96 | 3.2 | 244 | 1.8 | 204 |
| Kidney | 0.39 | 81 | 2.7 | 302 | 2.05 | 180 |
| Lung | 0.67 | 47 | 2.8 | 327 | 1.75 | 276 |
| Liver | 0.67 | 47 | 9.3 | 81 | 4.4 | 80 |
| Bladder[e] | <0.2 | 159 | 3.0 | 250 | 1.5 | 231 |
| Mesentery[f] | 0.45 | 71 | 0.8 | 1991 | 0.3 | 299 |

[a] $T_{pot} = \lambda T_S/LI$ with $T_S = 11$ h and $\lambda = 0.693$ for normal tissues and $= 0.74$ for tumours and placenta.

[b] From extrapolation through 1 h and 7 day labelling indices, using eq. 2 in text.

[c] From extrapolation through 1 h and 3 day labelling indices, using eq. 3 in text.

[d] Using 48 h value because 72 h value is anomalously low.

[e] Data from Stewart *et al.*, 1980.

[f] Data from Hirst *et al.*, 1980.

**Figure 1** Continuous labelling of endothelium in 5 types of experimental tumour. Tritiated thymidine was injected i.p. every 8 h, beginning when the tumours measured 3.5–5.0 mm mean diameter. Each point represents a separate animal.

**Figure 2** Continuous labelling of endothelium in larger experimental tumours, measuring 7–8 mm mean diameter at the time of the first injection. ([$^3$H]dT) was administered i.p. every 8 h.

**Table III** Endothelial proliferation in different types of vessels

| | *Arteries & arterioles* | *Veins & venules* | *Capillaries* |
|---|---|---|---|
| Present data | $\frac{3}{1162}=0.30\%$ | $\frac{36}{1207}=3.0\%$ | $\frac{29}{5197}=0.60\%$ |
| Spaet & Lejneiks (1967) | $\frac{28}{22{,}000}=0.13\%^{a}$ | $\frac{27}{7300}=0.37\%$ | $\frac{64}{10{,}600}=0.6\%$ |
| Compilation of published data[b] | $\frac{45}{1000}=0.45\%$ | — | $\frac{289}{239{,}567}=0.11\%$ |

[a]Includes estimates for aorta derived from count per field × number of fields × number of sections.
[b]Data from: Engerman (1967) Gaynor (1971) Hirst *et al.* (1980) Stewart *et al.* (1980) Tannock & Hayashi (1972)

**Figure 3** Uptake of tritiated thymidine into the endothelial cells of eight normal tissues, when administered by repeated i.p. injection every 8 h. Note the expanded vertical scale and the very low uptake. All 8 tissues show a slow rate of endothelial proliferation.

days. These data are grossly different from those for the other normal tissues and actually show a more rapid uptake of tritiated thymidine than any of the tumours studied. All of the endothelial cells were labelled within 4 days. This indicates that endothelial proliferation can occur even more rapidly in placenta than in transplanted tumours.

We also measured the endothelial labelling index in a selection of slides of human tumours and it was found to fall within the same range as that measured for rodent tumours. A short exposure of biopsy specimens to ([$^3$H]dT) *in vitro* resulted in 2.8–4.6% of the endothelium being labelled in assorted lymphomas and epithelial tumours (provided by Dr. J. Kummermehr). After administration of ([$^3$H]dT) *in vivo,* 4.2–8.1% of endothelial cells were labelled in a human glioblastoma (provided by Dr. T. Hoshino) and 10–20% in a human parotid tumour (provided by Dr. C. Nervi).

**Figure 4** Uptake of tritiated thymidine into the placenta when administered i.p. every 8 h from the 10th day of gestation. There is considerable scatter at the early intervals but endothelial proliferation is obviously rapid.

### Discussion

These data confirm our earlier finding of a large and consistent difference between the proliferation rate of endothelium in normal and tumour blood vessels (Denekamp, 1982; Denekamp & Hobson, 1982). The initial labelling indices 1 h after a pulse label differ on average by a factor of 26, and this large difference persists after continuous labelling for periods up to 7 days. The normal tissue values at 1 h ranged from 0.04–0.67% (brain and muscle *versus* liver); it was difficult to be certain of the statistical significance of these differences because of the small number of labelled cells. However, the same order of difference is apparent after 7 days labelling, when the brain still shows very little uptake of ([$^3$H]dT), whereas in the liver ~10% of the endothelial cells are labelled (Table II). Because of its function as a detoxifying organ the liver endothelium is exposed to a high concentration of potentially toxic metabolites. Furthermore, part of the afferent supply of blood is through the portal vein and is relatively poorly oxygenated. These factors may combine to lead to a more rapid death and replacement of endothelial cells in the liver blood vessels. In addition it is difficult to be absolutely sure that no Küpffer cells were included in the counts, although liver sinusoids were generally avoided.

The labelling in the five types of tumour is of a different order of magnitude. No significant differences were seen in small (3–5 mm) tumours compared with large tumours (Figures 1 & 2). The initial LI values of 3–14% rose rapidly for the first 2–3 days and then for some tumours more slowly over the remaining 4 days of the study. This change in rate of uptake of tritiated thymidine in 4/5 tumours suggests that not all of the endothelial cells in the tumour vessels are actively involved in proliferation. The change in slope at ~30% and 50% labelling could be interpreted as a growth fraction of 30 and 50% in these tumours (Steel *et al.*, 1966). However, it is not possible to deduce from these data whether 50–70% of the cells in *each vessel* are quiescent, or whether proliferation has completely ceased in this proportion of the vessels within each tumour. It seems more likely that the latter would be true: more active endothelial proliferation might occur in the vessels nearest to the outer rim of the tumour, since the tumour is expanding radially from the centre and the nutritional quality of the blood in deeper vessels is likely to be poor. An elevated labelling index has been reported for the rim of liver metastases, once they exceed 1 mm in diameter (Bassermann & Rabes, 1983). These authors also observed increased proliferation of the sinus endothelium in the immediately adjacent normal liver.

The labelling in the placenta increased most rapidly with continuous labelling over the first 48 h. There is a large spread of values from one animal to another for this tissue over this early period. This is consistent with other reports of large variations in ([$^3$H]dT) uptake in foetal and placental tissue, even for adjacent foetuses in the same mother (Atlas *et al.*, 1960). In spite of the scatter it is obvious that endothelial proliferation in the placenta is even more rapid than that in any of the five experimental tumours in the present study.

These labelling data can be used in several ways to estimate the potential doubling time ($T_{pot}$) of the endothelium. In order to calculate this parameter a value for the DNA synthesis phase $T_S$ has to be used. Korr *et al.* (1975) used a double labelling technique for endothelial cells in normal brain and derived an estimate of $11.0 \pm 2.2$ h for $T_S$. In principle the continuous labelling data in Figures 1–4 can also be used to estimate $T_S$ for each tumour and tissue (Yamada & Puck, 1961), but this gives an enormous spread of $T_S$ values (7 h–$\infty$). Korr's measured value of $T_S = 11$ h for endothelial cells in brain capillaries has therefore been adopted for the following calculations. Steel (1968) showed that $T_{pot}$ could be calculated for an expanding population from the initial labelling index after a single injection, using the formula

$$T_{pot} = \lambda \frac{Ts}{LI} \qquad (1)$$

where $\lambda$ is a factor which corrects for the non linear age distributions of cells around the cell cycle. A value of 0.693 for $\lambda$ seems appropriate for cells with a long cell cycle time, or a large fraction in $G_0$; this value has therefore been adopted for normal tissues, and $\lambda = 0.74$ for the 5 tumours and the placenta (Steel, 1977).

If all the cells in the population are in the growth fraction, the labelling index should reach 100% in a time corresponding to the potential doubling time minus $T_S$. This can be derived from the $LI_{1h}$ and $LI_{7\,day}$ values as follows:

$$T_{pot} - T_S = \frac{100\% - LI_{1\,h}}{LI_{7d} - LI_{1\,h}} \times 7 \text{ days} \qquad (2)$$

If only a proportion of the cells are in the growth fraction and these supply cells to the non-proliferating compartment there would be an initial rapid increase in LI until all the cells in the growth fraction have taken up label, and then a slower increase as the labelled cells gradually dilute out the non-dividing subpopulation. (Steel *et al.*, 1968). Thus, the very shallow slope for the normal tissues may result from a very small growth fraction of 1–2%, as has been suggested by Korr *et al.*, (1975) and Hirst *et al.* (1980). Therefore a third estimate of $T_{pot}$ has been made using the data at an earlier interval i.e. 2–3 days (Table II)

$$T_{pot} - T_S = \frac{100\% - LI_{1\,h}}{LI_{3d} - LI_{1\,h}} \times 3 \text{ days} \qquad (3)$$

The estimates from this latter equation are generally shorter than those from eq. (2), because they are derived from the initial steeper increase in labelling.

All these estimates of $T_{pot}$ are listed in Table II and are summarised in Figure 5. Two other published normal tissues studied at the Gray Laboratory have been included for comparison: bladder endothelium (Stewart *et al.*, 1980) and mesenteric arterioles (Hirst *et al.*, 1980). There is a considerable variation in the estimates of $T_{pot}$ from the three equations. However, there is clearly a very large difference between the normal adult tissues on the one hand and the placenta and tumours on the other (note the vertical axis in Figure 5 is on a logarithmic scale).

Among the unstimulated normal tissues the liver shows the shortest turnover time and the brain the longest. Within each tissue there may also be subtle differences in vessels of different calibre. In a limited analysis of veins, arteries and capillaries of normal tissues we observed the highest proliferation index in the veins (Table III). This is somewhat surprising in view of the hypothesis that endothelial cell turnover is most marked in regions of highest turbulence (Payling Wright, 1968), since flow is more rapid and pulsatile in arteries than in veins. However the endothelial cells in veins are probably subject to more stretch since the vessel diameter can vary more because of their less rigid wall structure. There is only one study in the literature where a systematic investigation of vessels of different calibre has been made (Spaet & Lejneiks, 1967). Their data indicated very little difference in different vessels, but the highest uptake of ([$^3$H]dT) occurred in capillaries (Table III). A review of all the other published data, sub-divided into different vessel types, is included in Table III. Most of the studies are stated to be of capillaries, in which almost a quarter of a million cells have been assessed. From this compilation of data the highest labelling index occurs in arteries and arterioles. Table III shows that no consistent picture emerges as to which type of vessels have the highest labelling index. This point is worthy of further study.

**Figure 5** Turnover times calculated from labelling indices (●) from $T_{pot}=T_S/LI_{1h}$; (■) using 1 h and 3 day labelling indices; (△) using 1 h and 7 day labelling indices.

Data from **Figures 1–4**, and 36 values for 1 h LI from Gunduz (1981), 10 values from Tannock (1970 and pers. comm.), 13 values from Denekamp & Hobson (1982) for tumours, plus data from Hirst *et al.*, (1980) for mesentery and Stewart *et al.*, (1980) for bladder. Nine human tumours were counted (by B.H.) from slides provided by Drs. Nervi, Kummermehr & Hoshino.

The high uptake of thymidine into the placenta indicates that if it is subject to an appropriate stimulus the endothelium is capable of even more rapid proliferation than that which occurs in the animal tumours we tested. Tumour angiogenesis factors have a large effect on the direction of capillary budding and endothelial cell migration, perhaps with increased endothelial cell proliferation as a secondary consequence (Folkman, 1983; Kumar, 1983). Capillaries within tumours, however, are surrounded on all sides by the angiogenic stimulus and may therefore be less profoundly influenced by TAF than vessels stimulated unilaterally in the model systems used to study TAF.

The present studies confirm the large difference previously reported between tumour and normal blood vessels, but with more statistical accuracy because of the continuous labelling. This provides a quantitative basis to the general observation of increased angiogenesis in tumours. The comprehensive study of tumour vascular proliferation in lung metastases by Gunduz (1981) showed an even higher labelling index than those reported here. Over a wide range of tumour sizes he found LI 1 h = 14% and he also saw a dramatic increase in LI with repeated injections. His data differed by a factor of 100 from those for normal tissues reported by Gaynor (1971), despite the statement to the contrary in his paper.

These data confirm that there is a sound basis for continuing to seek methods of attacking proliferating endothelium as a means of targeting cytotoxic treatment to tumours (Denekamp, 1982; Denekamp & Hobson, 1982).

We are grateful to C. Parkins, V. Randhawa, A. Rojas and N.H.A. Terry for help with the continuous labelling studies and to the Mount Vernon Hospital Pathology Department for processing and cutting the sections. We thank Prof J.F. Fowler for his continual constructive criticism, Mrs E.E. Marriot for secretarial assistance and Mr P. Russell and his staff for the production and care of the mice. This work was entirely financed by the Cancer Research Campaign.

### References

ATLAS, M., BOND, V.P. & CRONKITE, E.P. (1960). Deoxyribonucleic acid synthesis in the developing mouse embryo studied with tritiated thymidine. *J. Histochem. Cytochem.*, **8**, 171.

BASSERMANN, R. & RABES, H.M. (1983). Angiogenesis in experimentally induced metastases. *Verh. Dtsch. KrebsGes*, **4**, 853.

DENEKAMP, J. (1982). Endothelial cell proliferation as a novel approach to targetting tumour therapy. *Br. J. Cancer*, **45**, 136.

DENEKAMP, J. & HOBSON, B. (1982). Endothelial cell proliferation in experimental tumours. *Br. J. Cancer*, **46**, 711.

DENEKAMP, J. & STEWART, F.A. (1978). Evidence for repair capacity in mouse tumours relative to skin. *Int. J. Radiat. Oncol. Biol. Phys.*, **5**, 2003.

DENEKAMP, J. HIRST, D.G., STEWART, F.A. & TERRY, N.H.A. (1980). Is tumour radiosensitization by misonidazole a general phenomenon? *Br. J. Cancer*, **41**, 1.

ENGERMAN, R.L., PFAFFENBACH, D. & DAVIES, M.D. (1967). Cell turnover of capillaries. *Lab. Invest.*, **17**, 738.

FOLKMAN, J., MERLER, E., ABERNATHY, C. & WILLIAMS, G. (1971). Isolation of a tumour factor responsible for angiogenesis. *J. Exp. Med.*, **133**, 275.

FOLKMAN, J. (1983). The role of heparin in angiogenesis. In Development of the Vascular System. *CIBA Symp.*, **100**, 132.

GAYNOR, E. (1971). Increased mitotic activity in rabbit endothelium after endotoxin. An autoradiographic study. *Lab. Invest.*, **24**, 318.

GUNDUZ, N. (1981). Cytokinetics of tumour and endothelial cells and vascularisation of lung metastases in $C_3H/He$ mice. *Cell Tissue Kinet.*, **14**, 343.

HEWITT, H.B., BLAKE, E.R. & WALDER, A.S. (1976). A critique of the evidence for active host defence against cancer, based on personal studies of 27 murine tumours of spontaneous origin. *Br. J. Cancer*, **33**, 241.

HILL, S.A. & DENEKAMP, J. (1979). The response of six mouse tumours to combined heat and X-rays – Implications for therapy. *Br. J. Radiol.*, **52**, 209.

HIRST, D.G. & DENEKAMP, J. (1979). Tumour cell proliferation in relation to the vasculature. *Cell Tissue Kinet.*, **12**, 31.

HIRST, D.G., DENEKAMP, J. & HOBSON, B. (1980). Proliferation studies of the endothelial and smooth muscle cells of the mouse mesentery after irradiation. *Cell Tissue Kinet.*, **13**, 91.

HIRST, D.G., DENEKAMP, J. & HOBSON, B. (1982). Proliferation kinetics of endothelial and tumour cells in three mouse mammary carcinomas. *Cell Tissue Kinet.*, **15**, 251.

HOPEWELL, J.W. (1974). The late vascular effects of radiation. *Br. J. Radiol.*, **47**, 157.

KORR, H., SCHULTZE, B., & MAURER, W. (1975). Autoradiographic investigations of glial proliferation in the brain of adult mice. *J. Comp. Neur.*, **150**, 169.

KUMAR, S. (1983). Discussion (no title). In *Development of the Vascular System. CIBA Symp.*, **100**, 176.

PAYLING WRIGHT, H. (1968). Endothelial mitosis around aortic branches in normal guinea-pigs. *Nature*, **220**, 78.

RUBIN, P. & CASARETT, G.W. (1968). *Clinical Radiation Pathology* Vols. I & II. Saunders, Philadelphia.

SPAET, T.H. & LEJNIEKS, I. (1967). Mitotic activity of blood vessels. *Proc. Soc. Exp. Biol. Med.*, **125**, 1197.

STEEL, G.G., ADAMS, K. & BARRATT, J.C. (1966). Analysis of the cell population kinetics of transplanted tumours of widely differing growth rate. *Br. J. Cancer*, **20**, 784.

STEEL, G.G. (1968). Cell loss from experimental tumours. *Cell Tissue Kinet.*, **1**, 193.

STEEL, G.G. (1977). *Growth Kinetics of Tumours*. Oxford University Press.

STEWART, F.A., DENEKAMP, J. & HIRST, D.G. (1980). Proliferation kinetics of the mouse bladder after irradiation. *Cell Tissue Kinet.*, **13**, 75.

TANNOCK, I.F. (1970). Population kinetics of carcinoma cells, capillary endothelial cells and fibroblasts in a transplanted mouse mammary tumour. *Cancer Res.*, **30**, 2470.

TANNOCK, I.F. & HAYASHI, S. (1972). The proliferation of capillary endothelial cells. *Cancer Res.*, **32**, 77.

VAN DER KOGEL, A.J. & BARENDSEN, G.W. (1974). Late effects of spinal cord irradiations with 300 kV X-rays and 15 MeV neutrons. *Br. J. Radiol.*, **47**, 393.

YAMADA, M. & PUCK, T.T. (1961). Action of radiation on mammalian cells. IV. Reversible mitotic lag in the $S_3$HeLa cell produced by low doses of X-rays. *Proc. Natl Acad. Sci.*, **47**, 1181.